# Nebulizers effectiveness on pulmonary delivery of alpha-1 antitrypsin

**DOI:** 10.1007/s13346-023-01346-3

**Published:** 2023-04-25

**Authors:** Annalisa Bianchera, Viviana Vilardo, Roberta Giaccari, Annalisa Michielon, Gianluca Bazzoli, Francesca Buttini, Marina Aiello, Alfredo Chetta, Stefano Bruno, Ruggero Bettini

**Affiliations:** 1 Food and Drug Department, University of Parma, Parco Area Delle Scienze 27/a, Parma, Italy; 2 Interdepartmental Center Biopharmanet-Tec, University of Parma, Parco Area Delle Scienze Building 33, Parma, Italy; 3 Department of Medicine and Surgery, University of Parma, Via Gramsci 14, Parma, Italy

**Keywords:** Alpha-1 antitrypsin, Jet nebulizer, Vibrating mesh nebulizer, Protein drug delivery

## Abstract

The nebulization of alpha-1 antitrypsin (AAT) for its administration to the lung could be an interesting alternative to parenteral infusion for patients suffering from AAT genetic deficiency (AATD). In the case of protein therapeutics, the effect of the nebulization mode and rate on protein conformation and activity must be carefully considered. In this paper two types of nebulizers, i.e., a jet and a mesh vibrating system, were used to nebulize a commercial preparation of AAT for infusion and compared. The aerosolization performance, in terms of mass distribution, respirable fraction, and drug delivery efficiency, as well as the activity and aggregation state of AAT upon in vitro nebulization were investigated. The two nebulizers demonstrated equivalent aerosolization performances, but the mesh nebulizer provided a higher efficiency in the delivery of the dose. The activity of the protein was acceptably preserved by both nebulizers and no aggregation or changes in its conformation were identified. This suggests that nebulization of AAT represents a suitable administration strategy ready to be translated to the clinical practice for delivering the protein directly to the lungs in AATD patients, either as a support therapy to parenteral administration or for subjects with a precocious diagnosis, to prevent the onset of pulmonary symptoms.

**Graphical Abstract:**

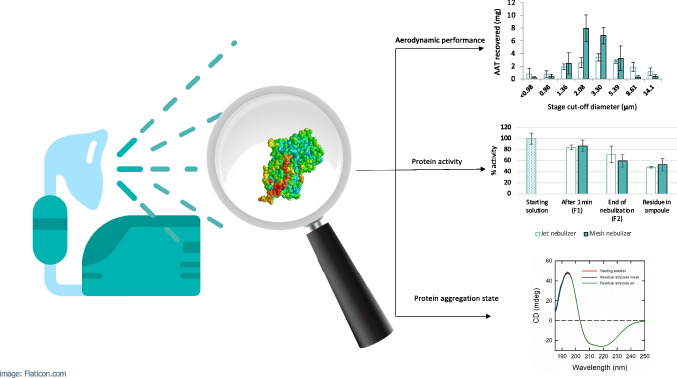

## Introduction

Alpha-1 antitrypsin deficiency (AATD) is a genetic disorder that predisposes affected individuals to liver and lung disease. AATD originates from mutations of the SERPINA-1 gene, which encodes alpha-1 antitrypsin (AAT), a glycosylated serpin mostly produced in the liver and one of the main components of plasma [1]. The liver manifestations of AATD mainly result from AAT misfolding, leading to the formation of intracellular aggregates. In contrast, the lung-related disease is associated with hypofunctional or non-functional AAT variants that insufficiently inhibit neutrophil elastase (NE), a serine protease that acts as an intracellular and extracellular microbicidal agent. A higher NE activity results in the proteolysis of pulmonary elastin, and, in the long term, in pulmonary emphysema and bronchiectasis [1].

The current therapeutic approach to AATD treatment consists of weekly slow intravenous administrations of AAT purified from human plasma to restore the protective plasma level of about 60 mg/dL [2]. Infusions are costly and require ambulatorial treatment and healthcare personnel. Moreover, despite the lungs being the organs mainly affected by AATD, only 3% of intravenously administered AAT is reported to reach the pulmonary epithelium [3]. Therefore, the local administration of AAT has been considered a potential alternative, if an appropriate amount of active AAT reaches the lower respiratory tract in the active form. This idea is not new in the field of AAT therapy: in the past, AAT has been administered by nebulization with jet nebulizers to healthy volunteers [4], cystic fibrosis patients [5], and AATD patients [6].

The products for nebulization currently on the market for chronic diseases are commonly prescribed with a recommended nebulizer since the inhaled dose can vary considerably depending on the nebulization system. In fact, inhalation medicines are true drug/device combination products. In the case of protein therapeutics, the effect of the nebulization mode and rate on protein conformation and activity should also be considered. Indeed, a protein therapeutic could denature—and therefore lose its activity—due to the interaction with the air–liquid interface, oxidation, thermal degradation, and mechanical stress [7]. These issues were addressed for AAT by Flament et al. [8, 9], who focused on the effects of formulation excipients and technological parameters on the nebulization by jet and ultrasonic nebulization systems. In these works, the addition of surfactants improved the respirable fraction of AAT, but the type of nebulizer and operating conditions played a major role. The advent of vibrating mesh nebulizers in the early 2000s—which eliminated the problem of solution recirculation—opened new possibilities for the efficient nebulization of drugs, particularly biopharmaceuticals. However, during nebulization, heating of the drug solution occurs, potentially affecting thermolabile proteins. Wild-type AAT, in particular, was reported to denature at a temperature of about 48 °C [10]. Moreover, protein aggregation by interfacial degradation was reported with these types of nebulizers [7, 11].

Research works and some clinical trials have described the administration of AAT to patients by nebulization to exploit its anti-inflammatory properties. Most of them involved CF patients [12–15]. All results agreed on the safety of this approach and provided evidence of its efficacy in reducing lung inflammation. On the other hand, when considering the inhibition of elastase activity in AAT patients [16–18], a high peripheral deposition of AAT was reported, but the clinical parameters did not provide conclusive evidence of elastase inhibition and improvement in pulmonary function, as reviewed elsewhere [19]. In our opinion, these results suffer from a limited consideration of the biological activity and aggregation of the protein upon nebulization, since the sole estimation of the dose of protein reaching the deep lungs is not sufficient to guarantee the desired therapeutic effect, particularly in consideration that all nebulization systems expose protein solutions to potentially damaging conditions.

To address these issues, in this paper we investigated not only the drug delivery efficiency but also the activity and aggregation state of AAT upon in vitro nebulization with two types of nebulizers, i.e., a jet and a mesh vibrating system as crucial element for the efficient translation of liquid formulations into effective therapeutic medicines for nebulization.

## Materials and methods

### Materials

Prolastin^®^ powder for infusion, containing 1000 mg of human plasma-purified AAT (Grifols, Barcelona, Spain, Batch G45BE00351) was divided into aliquots and dissolved in ultrapure water produced by an Arium^®^ purification system (Sartorius, Goettingen, Germany). A 25 mM potassium phosphate buffer solution was prepared by dissolving 2.336 g of K_2_HPO_4_ and 1.577 g of KH_2_PO_4_, both from A.C.E.F. (Fiorenzuola, Italy), in 1 L of ultrapure water. The Bradford assay kit was from Bio-Rad Laboratories (Milan, Italy). Porcine elastase was from Sigma-Aldrich (St. Louis, Missouri, USA). All other materials were from Sigma-Aldrich, unless otherwise stated.

### Nebulizers and nebulization procedure

Two commercially available inhalation systems were tested for the in vitro evaluation of nebulization of AAT: a breath-enhanced jet nebulizer system, TurboBOY^®^, equipped with a PARI LC Sprint^®^ ampoule, and an electronic active vibrating membrane nebulizer, the eFlow^®^ rapid apparatus, both from PARI GmbH (Munich, Germany). Adequate amounts of freeze-dried powder were weighed and dissolved under gentle stirring in ultrapure water to a final protein concentration of 25 mg/mL (2.5% w/v). Six mL of protein solution (i.e., 150 mg of AAT) were loaded in ampoules for nebulization both for the delivered dose and aerodynamic profile tests.

### Determination of delivered dose and drug delivery rate

The delivered dose (DD), representing the overall mass of protein emitted, the drug delivery rate (DDR), representing the mass emitted per minute, and the time needed to aerosolize the entire dose loaded in the ampoule, were determined following the specifications in the European Pharmacopoeia 11^th^ ed. [20]. A breathing simulator (sine pump Model SRV500CC, VCS Srl, Parma, Italy) was set to mimic an adult breathing pattern of 15 breaths/min, with a tidal volume of 500 mL per inspiration. The inhalation:expiration ratio was 1:1. For both nebulizers, the mouthpiece of each ampoule was connected to the sine pump by a specific rubber adaptor and through a filter holder (PARI Pharma GmbH, Munich, Germany) equipped with a filter (type A/E glass diameter 76 mm, Pall Corporation, New York, US) to entrap the nebulized aerosol. For DDR, the nebulizer was run for 1 min; thereafter, the filter, coded as F1, was removed, and substituted with a new filter (F2), then the nebulizer was activated and run until the end of the nebulization. Filter F2 was kept for a maximum of 5 min, then substituted with a new filter (F3) to prevent breakage and overload. For the jet nebulizer, the end of nebulization was set 1 min after hearing sputtering; for the mesh nebulizer, the end of nebulization was determined by its automatic stop.

To estimate the amount of AAT deposited on the filters, each of them was transferred into a glass crystallizer and washed in ice-cold 25 mM phosphate buffer for 5 min to recover the nebulized protein. Thereafter, the solution was filtered through a cellulose acetate membrane with a pore size of 0.45 μm, transferred in a 10-mL flask, brought to volume with cold phosphate buffer, and stored on ice until the concentration of AAT was determined.

The DDR was estimated by quantifying the mass of AAT collected by F1, while DD was determined as the sum of the mass collected by all filters. The efficiency of the procedure for the protein recovery from filters was estimated by quantifying with Bradford assay (see below) the amount of AAT collected from a filter imbibed with 0.5 mL of AAT solution (25 mg/mL) and recollected in 10 mL of cold phosphate buffer.

Each test was carried out in triplicate.

### Determination of mass distribution and respirable fraction of AAT by next generation impactor

The mass distribution of AAT emitted by the two nebulizers was analyzed by Next Generation Impactor (NGI, Copley Scientific, Nottingham, UK). The apparatus was pre-cooled at 4 °C to prevent droplet evaporation and determinations were performed within 5 min of the removal of the impactor from the refrigerator. A continuous aspiration flow of 15 L/min was obtained by connecting the apparatus to a pump (VP1000, Erweka, Italy) governed by a Critical Flow Controller (TPK, Copley Scientific, Nottingham, UK). Each ampoule filled with the appropriate amount of protein solution was connected to the induction port of the apparatus by a rubber adaptor. The nebulizer was activated for 4 min, as prescribed in European Pharmacopeia 11^th^ edition [20], or for 2, 6, or 9 min, or until exhaustion of the solution in the ampoule, to assess the consistency of nebulization in time. The induction port, each stage, and the filter of the Micro Orifice Collector were rinsed with ice-cold phosphate buffer and each sample was collected and brought to volume in 20-mL volumetric flasks. The concentration of protein in each sample was quantified by Bradford protein assay [21] and the resulting amount (sum of the quantity recovered in each stage) used to calculate the emitted dose. The cumulative undersize mass percentage of AAT found in each stage was used to build a mass distribution plot with respect to the cut-off diameters of each stage: in particular, the median mass aerodynamic diameter (MMAD) and the geometric standard deviation (GSD) were calculated from the plot of cumulative undersize percentage of the collected drug (in probit scale) with respect to the log cut-off values of each stage according to European Pharmacopoeia specifications (2.9.18, 11^th^ ed.) [20], built with Microsoft Excel for Mac (ver. 16.57). This plot also allowed the determination of the fine particle fraction (FPF%), corresponding to the percentage mass of protein emitted in droplets having an aerodynamic diameter lower than 5 μm. This value was also used to estimate fine particle dose, namely the milligrams of proteins expected to reach the lower respiratory airways, as a product between the delivered dose, derived from DD/DDR experiments, and FPF%. Each test was carried out in triplicate.

### Protein quantification

The amount of AAT in each sample was estimated by UV–visible absorption spectroscopy at 280 nm using a Cary4000 spectrophotometer (Agilent Technologies, Santa Clara, USA), by Bradford assay (Bio-rad Laboratories, Milano, Italy) [21] or by 12% SDS electrophoresis (SDS-PAGE) [22] followed by densitometric analysis of the AAT band using a Bio-rad system. The Bradford assay was performed with a Spark 10 M microplate reader (Tecan, Mannedorf, Switzerland) as per manufacturer’s instructions. Each measurement was carried out in triplicate.

### Determination of AAT activity

The samples deriving from DD/DDR tests or NGI tests were snap-frozen in liquid nitrogen and then stored at −80 °C to prevent activity loss over time [23]. They were then thawed immediately before the activity assays were performed. AAT activity was assayed for each sample and normalized to its quantity, as determined by densitometric analysis of SDS-PAGE electropherograms. Briefly, AAT was incubated at 5 nM concentration, as determined by SDS-PAGE, with 10 nM porcine elastase in a solution containing 0.1 M HEPES, 0.5 M NaCl, and 0.05% Triton, pH 7.4 at 37 °C for 45 min. The residual elastase activity was determined by following the hydrolysis of N-succinyl-Ala-Ala-Ala-p-nitroanilide to *p*-nitroaniline at 410 nm in a Cary 4000 UV–Vis spectrophotometer. The fractional AAT residual activity (*A*) was calculated from the initial velocity of the elastase assay in the absence (*V*_ctrl_) and presence (*V*_*I*_) of AAT, according to Eq. 1:

1 $$A=1-(2\frac{{V}_{\mathrm{ctrl}}-{V}_{I}}{{V}_{\mathrm{ctrl}}})$$

Protein precipitation was assessed by SDS-PAGE as previously described [23]. All the measurements were carried out in replicate.

### Evaluation of protein alteration upon nebulization

Protein aggregation and structural integrity upon nebulization were evaluated by dynamic light scattering (DLS), size exclusion chromatography (SEC), and circular dichroism (CD). To prevent any protein alteration ascribable to its recovery from filters, as in DD/DDR experiments, these tests were performed on samples collected by nebulizing the solutions in a Twin Stage Impinger, according to European Pharmacopoeia specifications (2.9.18, 11^th^ ed.) [20] using a continuous aspiration flow of 60 L/min. Briefly, 7 or 30 mL of 25 mM phosphate buffer were introduced in the upper and lower impingement chambers. The nebulizers were run until sputtering (for jet nebulizer) or after the automatic stop of the device (for mesh nebulizer). Each sample from the upper impingement chamber was collected and brought to a final volume of 10 mL, while each sample from the lower impingement chamber was collected and brought to a final volume of 50 mL with potassium phosphate buffer*.* The solutions were immediately tested by dynamic light scattering, size exclusion chromatography, and circular dichroism.

### Dynamic light scattering, DLS

The starting protein solution and the residual solutions in the ampoules were collected and quantified. To perform DLS analysis, protein concentration was optimized by comparing the signals obtained from samples as such or after dilution in 10 mM NaCl aqueous solution. When no difference was observed between the original sample and its diluted counterpart, the measurement of the sample as such was considered (e.g., for samples deriving from impingement chambers), since no interaction was supposed to occur between molecules in solution; otherwise, the diluted sample was considered, as in the case of those deriving from ampoule, which were diluted 1:10 in 10 mM NaCl aqueous solution. Measurements were performed with a Zetasizer Nano (Malvern Instruments, UK) equipped with a 633-nm laser, using NIBS detection (173° backscatter) at 25 °C. Samples were analyzed immediately after they were obtained, without any prolonged storage or freezing step to avoid time- and temperature-dependent aggregation. Three measurements were performed for each sample, and considered as valid if the intercept of the correlation function was between 0.8 and 1. The three replicates were averaged to calculate the mean size and polydispersity index.

### Size exclusion chromatography, SEC

Chromatographic separation was achieved on a Prominence HPLC system coupled with a UV detector and LabSolutions software (Shimadzu, Kyoto, Japan) using a BioSep-SEC-2000 column (300 mm, 1.50 mm, 5 μM Phenomenex, Torrance, CA, USA) in isocratic elution mode with a mobile phase composed of 50 mM K_2_HPO_4_, 300 mM NaCl pH 7 and pumped at a flow rate of 1 mL/min. The injection volume was 50 μL at an estimated protein concentration of 1 mg/mL. The detection was performed with an SPD-20A Model UV detector (Shimadzu Kyoto, Japan) at 220 nm. Samples were centrifuged for 30 min at 16,000 × g before loading. Each chromatographic run lasted 15 min. The size of the aggregates was estimated by means of a calibration curve built with a Column Performance Check Standard, Aqueous SEC 1 (Phenomenex), including myoglobin (17 kDa), ovalbumin (44 kDa), IgG (150 kDa), IgA (300 kDa), and bovine thyroglobulin (670 kDa).

### Circular dichroism, CD

CD spectra were collected using a Jasco J-1500 spectropolarimeter (Jasco, Tokyo, Japan) equipped with a Peltier thermostatic unit set at 20 °C using 0.1 mm optical length quartz cells and driven by a JASCO Spectra Manager II software. The protein concentration was 5 µM in 10 mM K_2_HPO_4_ buffered at pH 7. The spectral scans were collected between 250 and 180 nm, 0.5 nm data pitch, 8 s DIT, bandwidth 2 nm, at 50 nm/min scanning speed. Each spectrum was the result of 3 averaged accumulations. Secondary structure estimation was performed by using the Dichroweb server [24]. All CD spectra were corrected for buffer background. Far‐UV CD signal changes at 220 nm were monitored as a function of increasing temperature from 20 to 90 °C, with steps of 5 °C and with an equilibration time of 1 s at each temperature before recording the measurement. The thermal transitions were analyzed with the CalFitter algorithm [25].

### Statistical analysis

Data were analyzed with Microsoft Excel for Mac using a *t*-test. Statistical significance was assumed at *p* < 0.05. If not otherwise specified, measurements were carried out in triplicate and represented as mean value and standard deviation.

## Results and discussion

### Preliminary assessment of dose and potential administration regimen

In this work, we investigated the use of nebulized AAT as an alternative, or at least as a support, to parenteral infusion. Medicines for inhalation are combination products whose performance, in terms of delivered dose and fraction of the dose reaching the targeted deep lung, is strongly affected by the proper combination of formulation and device. As for the suitable dose, an assessment of the potential amount of AAT to be administered was preliminarily required. The protective threshold of AAT in the epithelial lining fluid (ELF) is reported as 1.3–1.7 μM [26], although the level in healthy subjects is about 3 μM (0.15 mg/mL—lower threshold 0.07 mg/mL). Since the volume of ELF is estimated between 10 and 30 mL [27], the mass of *active* protein that should reach the deep lung is between 1.5 and 4.5 mg (lower threshold 0.7–2.1 mg). Considering the potential loss of protein during nebulization, and that part of the solution (about 1 mL) remains in the ampoule at the end of nebulization, we set a target dose of 150 mg. This dose is almost double the dose administered in other clinical trials (NCT01217671 and NCT04204252 [18, 28]), but this should not constitute a safety concern since up to 500 mg/day of recombinant AAT (rAAT) were previously administered to patients without signs of allergic reaction [14], and 100 mg of hAAT were administered twice a day for 1 week, also with no adverse effects [29]. The AAT concentration that we chose was 25 mg/mL, which corresponds to that of Prolastin^®^ upon reconstitution in water, as per the manufacturer’s instructions. This choice would allow the use of the commercial product off-label, without modifying the final concentration of the salts and excipients present in the lyophilized product. Indeed, hypertonicity could induce adverse reactions [30] such as bronchoconstriction [31] and cough [32], thus reducing the effectiveness of the deposition in the lungs. Given a concentration of 25 mg/mL, the proposed dose of 150 mg can be reached through a single nebulization of 6 mL, which also corresponds to the maximum capacity of the ampoules of the two nebulizers.

### Evaluation of the aerosolization performance of nebulizers

We measured the effect of the two nebulizers on the size distribution of the droplets obtained from 25 mg/mL AAT solutions with 4 min of nebulization (Table 1). MMAD values were comparable, despite a higher GSD for the jet nebulizer. In particular, MMAD for the jet nebulizer (3.9 µm) was in agreement with the producer’s specifications and was consistent with the AAT values reported by Griese et al. (3.5 µm) [15] and Hatley et al. (3.67 ± 0.72 µm by laser diffraction) [33], and not statistically different from the ones reported by Brand et al. (4.4 ± 2 μm) [16]. The apparent difference with the latter could be ascribed either to the different type of impactor (an eight-stage cascade impactor, instead of an NGI, which is considered more efficient [11]) or to the different AAT concentration used in that study (50 mg/mL AAT vs. 25 mg/mL), although increased viscosity, due to higher protein concentration, would be expected to decrease droplet size [34, 35]. The presence of salts in Prolastin^®^ after reconstitution should also be taken into consideration and could be partially responsible for the reduction of MMAD [34]. This effect could also explain the MMAD upon nebulization with the mesh nebulizer (3.3 μm), which was lower than the one reported in the producer’s specifications (4.1 μm), as well as the values reported by Hatley (4.4 μm), though measured by laser diffraction on salbutamol solutions [33], and by Brand et al. (4 ± 1.6 μm), with an analogous vibrating mesh device (AKITA^2^ APIXNEB) on radiolabelled Prolastin^®^ solution at 35 mg/mL [17]. Finally, the fine particle fraction of both nebulizers was significantly higher than previously reported by Hatley et al. (67.5 ± 1.0% for the jet nebulizer and 59.9 ± 3.7% for the mesh nebulizer) [33] and Brand et al. (67–70% for AKITA^2^ APIXNEB) [17].

**Table 1:** Mass median aerodynamic diameter, geometric standard deviation, and fine particle fraction obtained from the two nebulizers evaluated after 4 min by NGI (mean ± standard deviation; *n* = 3)

	**Jet nebulizer**	**Mesh nebulizer**
MMAD (μm)	3.9 ± 0.1	3.3 ± 0.3
GSD (μm)	2.5 ± 0.5	1.7 ± 0.1
Fine particle fraction (%)	71.8 ± 2.4	78.2 ± 1.5

The fine particle fraction produced by the mesh nebulizer was significantly higher than that obtained with the jet nebulizer (*t*-test, *p* < 0.001).

**Fig. 1:**
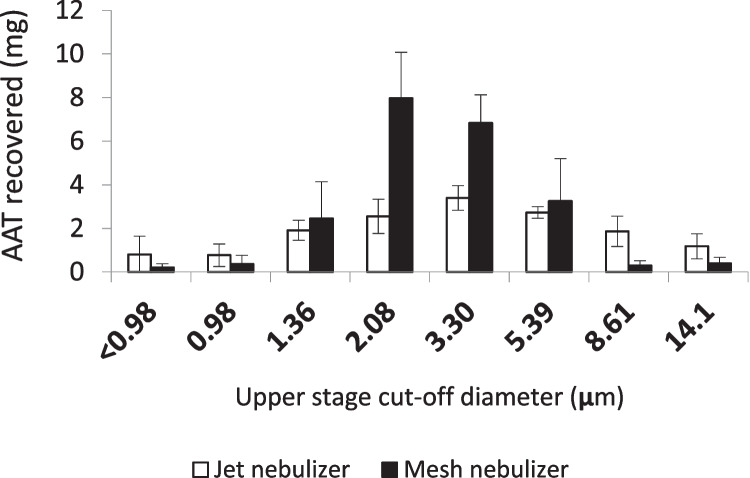
Mass distribution of AAT obtained after nebulization of the AAT solution in the NGI with the jet nebulizer and the mesh nebulizer. The bars represent the standard deviation (*n* = 3)

This difference was due to the fact that most of the droplets emitted by the mesh nebulizer accumulated in stages 3–6 of the NGI having a cut-off between 5.39 and 1.36 μm, at 15 L/min (Fig. 1), and this could be ascribed to the positive effect exerted by the mesh membrane that contributed to the production of smaller droplets. This result is in good agreement with data reported by Brand et al. with AKITA^2^ APIXNEB [17]. An analogous difference was observed between the same two nebulizers when 160 mg colistimethate sodium were aerosolized (71.9% mesh vs. 59.7% jet) [36].

**Fig. 2:**
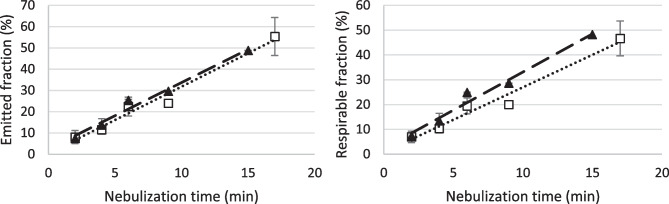
Emitted fraction (left) and respirable fraction (right) of AAT after nebulization of the solution with the jet nebulizer (empty square) and the mesh nebulizer (solid triangle) in NGI. The bars represent the standard deviation (*n* = 3). The lines are the graphical representation of the linear regression of the experimental data. Emitted fraction jet nebulizer *y* = 3.1429x + 0.381 (*R*^2^ = 0.9727) dotted line; mesh nebulizer *y* = 3.0351x + 2.5272 (*R*^2^ = 0.9662) dashed line; respirable fraction jet nebulizer y = 2.6202x + 0.7664 (*R*^2^ = 0.9678) dotted line; mesh nebulizer *y* = 2.9912x + 2.372 (*R*^2^ = 0.9634) dashed line

By prolonging the nebulization time, both nebulizers showed a linear relationship between emitted fraction as well as in fine particle fraction, and nebulization time (Fig. 2). The slope of the obtained regression lines was similar for the two nebulizers. Thus, no reduction in the efficiency of nebulization was observed, differently from what was reported by Steckel and Eskandar for a jet nebulization system analogous to the one used in these tests [37]: in that work, the decrease of efficiency was attributed to the increase of the concentration of the solution in the ampoule stemming from solvent evaporation. This aspect was investigated in the present work by sampling the undiluted nebulized product collected in pre-cooled 50-mL test tubes: samples were centrifuged before quantitation to remove any insoluble aggregate deriving from protein denaturation. No visible aggregates were detected, nor variation of the original concentration of protein solution, as assessed by UV absorbance, for any of the samples collected from the two nebulizers.

Nebulization time did not affect droplet size distribution, as no shift occurred in MMAD, for both types of nebulizers. However, the breadth of the distribution was smaller when using the mesh nebulizer, as reflected by the lower values of GSD (Table 2).

**Table 2:** Mass median aerodynamic diameter and geometrical standard deviation obtained from the two nebulizers after 2, 4, 6 min or at the end of nebulization by NGI (mean ± standard deviation; *n* = 3)

**Time (min)**	**Jet nebulizer**		**Mesh nebulizer**	
	**MMAD**	**GSD**	**MMAD**	**GSD**
**2**	3.3 ± 1.2	2.70 ± 0.01	3.2 ± 0.3	1.6 ± 0.2
**4**	3.9 ± 0.1	2.5 ± 0.5	3.3 ± 0.3	1.7 ± 0.1
**6**	3.1 ± 0.5	3.4 ± 0.1	3.1 ± 0.1	1.6 ± 0.1
**9**	3.9 ± 0.1	2.8 ± 0.1	3.2 ± 0.1	1.6 ± 0.1
**End**	3.3 ± 0.5	2.4 ± 0.5	3.1 ± 0.2	1.7 ± 0.1

### Determination of DDR and DD

To assess DDR and DD, ampoules were filled with 6 mL of AAT solution, and nebulization was performed by connecting the nebulizer to a sine pump (Table 3). Particular attention was paid to protein recovery from the collection filters to avoid denaturing conditions. This is a critical step, as previously reported [11]. Therefore, filters were not subjected to sonication to extract the protein, a cold phosphate buffer was used as a diluent, and all samples were centrifuged and processed in the shortest time possible. In these conditions, the efficiency of mass recovery was estimated at 98 ± 7%.

**Table 3:** Delivery values of AAT solutions using the two nebulizers (mean ± standard deviation; *n* = 3)

	**Jet nebulizer**	**Mesh nebulizer**
*DDR (mg/min)*	2.8 ± 1.2	2.8 ± 0.4
*DD (mg)*	35.0 ± 3.4	51.0 ± 8.5
*Estimated FPD (mg)*	25.1 ± 3.3	39.9 ± 7.4
*Nebulization time (min)*	17 ± 3	15 ± 3

By combining the value of the respirable fraction obtained by NGI with the DD, the fine particle dose was estimated at 25.1 mg for the jet nebulizer and 39.9 mg for the mesh nebulizer, respectively. The DDR, as assessed according to pharmacopeial specifications, was comparable between the two nebulizers, but the total drug delivered was significantly higher when using the mesh nebulizer (*p* = 0.033 by *t*-test). Therefore, the latter was more efficient than the jet nebulizer in delivering the solution, also considering that the time to complete nebulization was lower and that about 1 mL of the loaded solution was left over in the ampoule due to the automatic stop of the mesh device.

### Evaluation of AAT activity upon nebulization

**Fig. 3:**
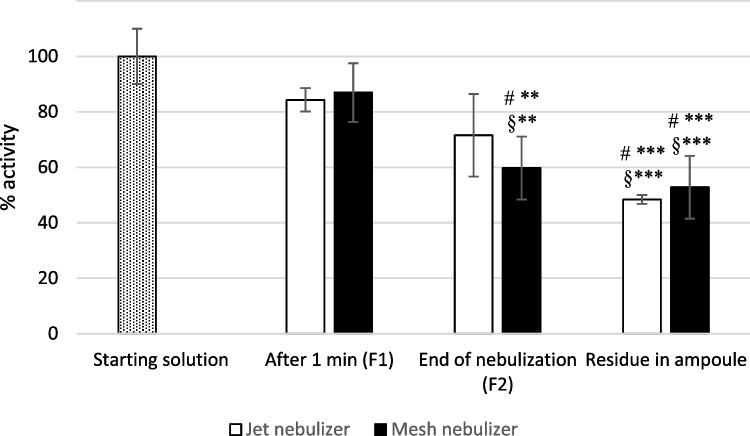
Activity of AAT obtained after nebulization of the solution with the jet nebulizer and the mesh nebulizer in comparison with the starting protein activity (dotted). # with respect to starting solution; § with respect to F1; **p < 0.01, ***p < 0.001

When dealing with inhaled products, three doses are commonly defined: the dose on the label (metered), the dose released by the device, and the dose that reaches the deep lung. If the active ingredient is a protein, a fourth dose value must also be considered, i.e., the dose that reaches the deep lung in a still biologically active form. Therefore, we measured the specific elastase inhibition activity of soluble AAT (i.e., the activity divided by the amount of protein measured in each fraction obtained from DD/DDR experiments) to evaluate possible inactivation due to oxidation or denaturation upon nebulization. The relative AAT activity was expressed as a percentage of that measured immediately after resuspension. As reported in Fig. 3, the activity of the protein tended to decrease with increasing nebulization time, with a comparable trend for both nebulizers. No significant differences could be detected between the two nebulizers at the same time points. After 1 min of nebulization, the activity was not significantly different (*t*-test) from that of the starting solution with both nebulizers, being 84% (± 4%) for the jet nebulizer and 87% (± 11%) for the mesh nebulizer. The slight reduction of activity could be at least partially ascribed to the procedure of recovery from the filter, that cannot be discriminated from the effect of nebulization.

Anyway, after the same recovery procedure, at the end of nebulization with the jet nebulizer, the activity decreased to 72% (± 15%), not significantly different from that of the starting solution, while with the mesh nebulizer, the final activity was 60% (± 11%) statistically lower than the starting solution, pointing at the nebulization procedure as the main cause of loss of activity. For both nebulizers, the AAT activity in the residual solution in the ampoule was significantly lower with respect to both the starting solution and the final nebulized product: this is probably ascribable to the fact that the solution in the ampoule is repeatedly stressed. The decrease in activity over time could either be associated with denaturation or to methionine oxidation.

To our knowledge, this is the first paper reporting data on AAT activity upon both jet and mesh nebulization. The effect of both nebulizers on protein integrity is in agreement with a paper on the nebulization of insulin-like growth factor I, which showed comparable performances by using jet or mesh nebulizers [38]. In both cases, a reduction in the activity of the protein was observed with respect to the starting solution. In this sense, the mesh nebulizer does not seem to provide a better preservation with respect to the jet nebulizer. The data relevant to the latter differ from what previously reported about the residual activity of AAT (95 ± 11.3%) after nebulization with eFlow^®^ [39, 40]. It is worth underlining that the conditions at which protein solution is exposed during DD/DDR test should be regarded as a worst-case scenario, considering the continuous air exposure and the collection on a solid dry support.

### Evaluation of AAT aggregation upon nebulization

**Fig. 4:**
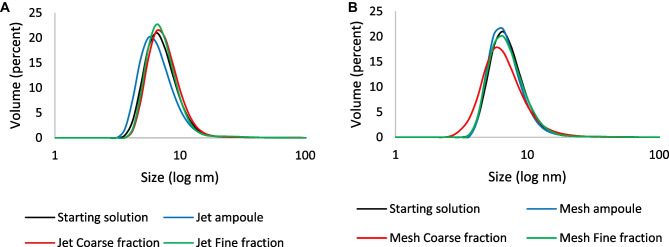
Size distribution analysis by volume of AAT dissolved in water before and after nebulization with jet (**A**) or mesh (**B**) nebulizers deriving from Twin Stage Impinger experiments

To ascertain the cause of the partial loss of specific activity over time, we evaluated potential protein aggregation of AAT before and after nebulization by means of DLS. Studying protein stability directly in the aerosol would be ideal but unfeasible. Among the apparatuses proposed by European Pharmacopoeia to assess aerodynamic performances of nebulized products, we selected the Twin Stage Impinger. The choice was driven by two main reasons: the first is that the collection of the nebulized product directly in liquid, in our opinion, better represents the wet environment found in the lungs by nebulized droplets; the volume of epithelial lung fluid is estimated between 10 and 30 mL [27], a volume that is close to the volumes prescribed by Eur. Ph. to fill the impingement chambers. The second reason is that the use of an impinger rather than an impactor could reduce the interferences in the protein state due to prolonged exposition to air and, specifically for NGI, to the contact with the metal components or, in DD/DDR tests, to the recovery from filters. DLS analysis of samples collected in impingement chambers indicated that, right after dissolution, the AAT solution presented a single population of macromolecules having a hydrodynamic diameter of 7 nm, broadly consistent with previous reports on monomeric, monodispersed AAT [23, 41]. The graph in Fig. 4 shows the size up to 100 nm to highlight any difference in the samples. Nebulization with both nebulizers did not modify the aggregation state of the protein, as no other populations appeared in ampoules as well as in fractions collected in the stages of the Twin Stage Impinger.

**Fig. 5:**
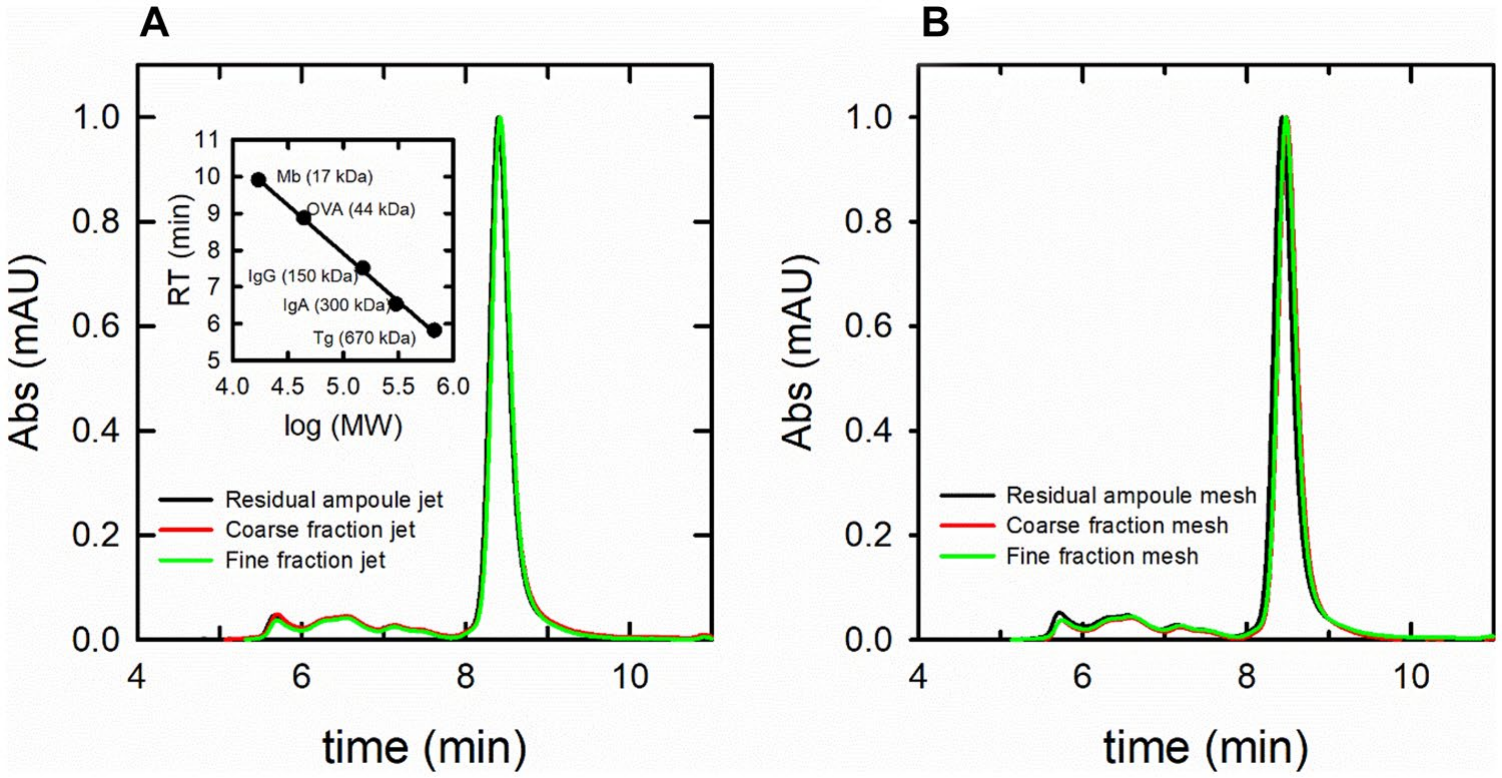
**A** SEC analysis of samples deriving from nebulization with the jet nebulizer compared with the starting solution. **B** SEC analysis of samples deriving from nebulization with the mesh nebulizer compared with the starting solution. The insert of panel **A** shows the calibration curve used to estimate the molecular weights. The band with elution time around 8.5 min correspond to a protein of 54 kDa consistent with glycosylated AAT

SEC analysis—which, in our configuration was sensitive in the 17–670 kDa MW range (up to 15-mers of AAT)—indicated a major band with an elution time around 8.5 min consistent with monomeric, glycosylated AAT (around 54 kDa) (Fig. 5). Multiple low-intensity bands with elution times of 5–7 min were consistent with aggregates with MWs ranging from 150 to 670 kDa. The chromatograms of nebulized and non-nebulized AAT were essentially superimposable for both mesh and jet nebulizers, confirming that nebulization did not affect aggregation. Moreover, in our tests, we could not find any evidence of an increase in the concentration of the solution ascribable to nebulization by both jet or mesh nebulizers, differently from what was reported by Steckel for jet nebulizers [37]. Overall, aggregation did not seem to be the cause of loss of activity for AAT, leaving methionine oxidation over time as the most likely cause of inactivation.

### Evaluation of AAT denaturation upon nebulization

**Fig. 6:**
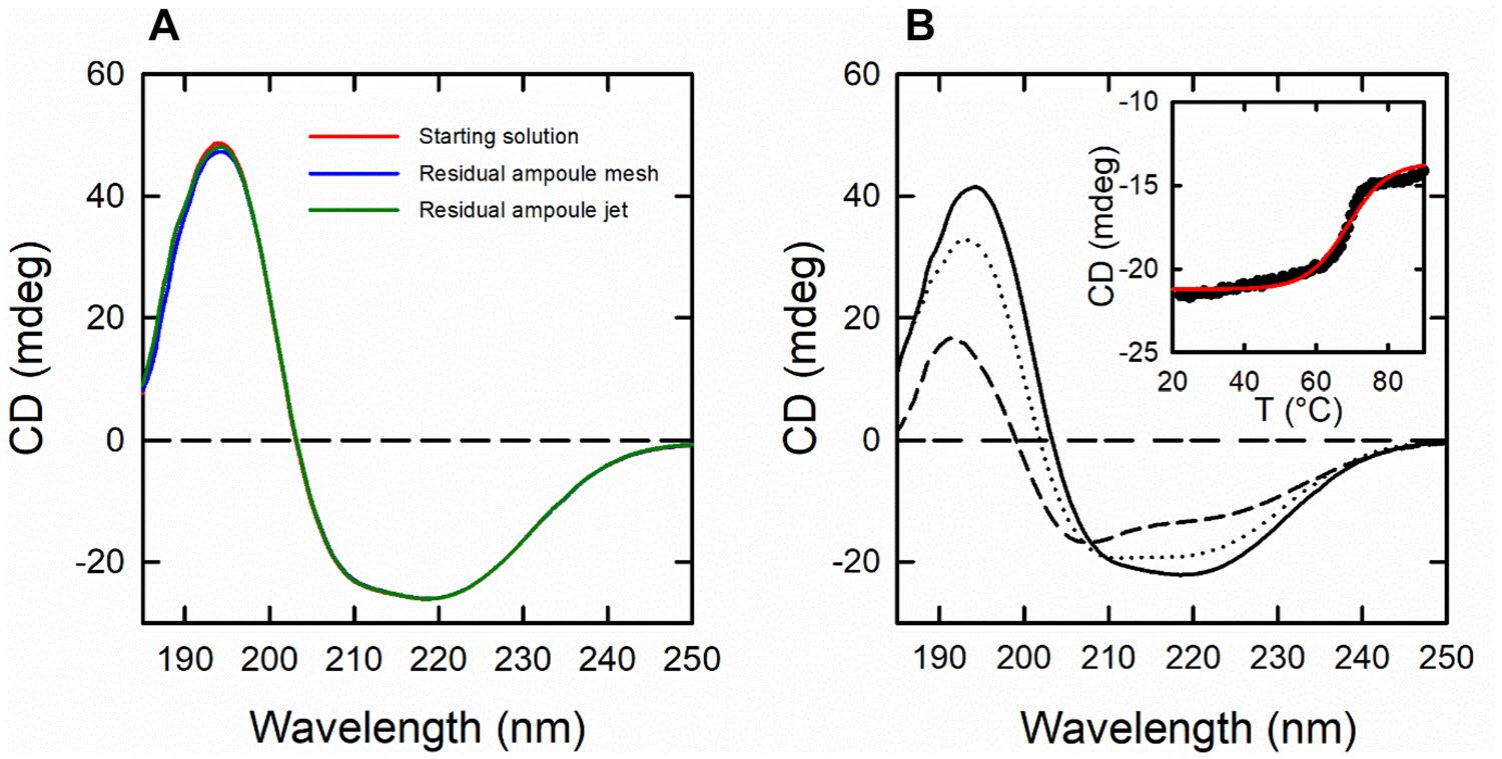
**A** CD analysis of samples deriving from nebulization with jet and mesh nebulizers compared to starting solution. **B** Circular dichroism spectra of AAT (solid line), AAT upon incubation at 90 °C (dashed line) and after incubation at 90 °C and slow return to 20 °C (dotted line). Inset: temperature ramp, with a calculated Tm of 69 °C

As temperature is known to affect protein stability, it was monitored in the ampoule before and after nebulization. A significant increase (+ 10.0 °C ± 3.1 °C) was observed with the mesh nebulizer, whereas a non-significant increase (+ 0.4 °C ± 2.3 °C) was observed in the ampoule of the jet nebulizer. To assess whether these temperature changes might be responsible for protein denaturation, and thus a partial loss in its specific activity, we measured CD spectra of the protein before and after nebulization. As reported in Fig. 6A, the spectra were completely superimposable.

Secondary structure analysis using the Dichroweb server confirmed almost identical structures (Table 4).

**Table 4:** Fraction of secondary structure content as evaluated with the dichroweb server. Each value is the average of the analysis of two independent preparations

	Helix 1	Helix 2	Strand 1	Strand 2	Turns	Unordered
AAT starting solution	0.1865	0.1205	0.1335	0.098	0.214	0.247
Mesh nebulizer ampoule	0.204	0.127	0.127	0.0955	0.207	0.2395
Jet nebulizer ampoule	0.17	0.115	0.145	0.099	0.2155	0.2555

To confirm that the temperature to which AAT is exposed during nebulization does not affect its folding, we performed thermal denaturation experiments monitored by CD at 220 nm. A transition temperature (T_m_) of 69 °C (Fig. 6B, inset) was calculated. However, the transition associated with this T_m_ was not associated with full loss of secondary structure, as indicated by CD spectra consistent with partial secondary structure preservation up to 90 °C (Fig. 6B). Moreover, a CD spectrum almost identical to that of the starting solution of AAT was obtained upon heating to 90 °C followed by slow cooldown, indicating that the secondary structure changes are reversible (Fig. 6B).

The exclusion of AAT precipitation, aggregation, and thermal denaturation in the ampoule suggested that the decrease in specific AAT activity of up to 50% was likely associated with the oxidation of AAT, particularly at residues Met358 and Met351 [42]. Oxidation of the protein is expected to occur to a greater extent when nebulized by jet nebulizer, due to recirculation of protein solution, which leads to repeated exposure of the protein to the air–liquid interface. For this reason, the residual in the ampoule has been considered a worst case for jet nebulization [43], and a consistent reduction in activity was expected. On the other hand, the stability of the residual solution in the ampoule of the mesh nebulizer in principle should not represent a major issue since it does not recirculate [7]. Differently from what expected, residual AAT activity in the ampoule of the mesh nebulizer was low and comparable to that in the ampoule of the jet nebulizer, hinting again at oxidation as the main degradation mechanism during nebulization.

Overall, no advantages were observed by using the jet nebulizer instead of the mesh nebulizer. The time required to complete the nebulization was not significantly different between the two nebulizers, being 17 ± 3 min for the jet nebulizer, as determined by hearing the typical sputtering sound, and 15 ± 3 min for the mesh nebulizer, defined by the automatic stop of the device. However, considering the higher dose delivered by the mesh nebulizer in the same nebulization time, the latter is overall more advisable in terms of efficiency.

## Conclusions

The results above provide a characterization of AAT activity after nebulization. Both nebulizers guarantee comparable and acceptable preservation of the activity of the nebulized protein and did not induce aggregation or changes in its conformation. This is of particular importance given the possible immunogenicity determined by aggregated proteins. The two nebulizers demonstrated equivalent aerosolization performance except for the fact that, with respect to the jet nebulizer, the mesh nebulizer provides higher efficiency in delivering the dose. This allows us to conclude that nebulization of a solution, even though not optimized for this administration way, may represent a suitable administration strategy in delivering the protein directly to the lungs in AATD patients, either as a support therapy to parenteral administration or, for subjects with a precocious diagnosis, to prevent the onset of pulmonary symptoms.

## Data Availability

The datasets generated during and/or analyzed during the current study are available from the corresponding author on reasonable request.

## References

[1] Kelly E, Greene CM, Carroll TP, McElvaney NG, O’Neill SJ (2010). Alpha-1 antitrypsin deficiency. Respir Med.

[2] Bianchera A, Alomari E, Bruno S. Augmentation Therapy with alpha-1 antitrypsin: present and future of production, formulation, and delivery. Current Medicinal Chemistry. 29:385–410. 10.2174/0929867328666210525161942 34036902

[3] Hubbard RC, Sellers S, Czerski D, Stephens L, Crystal RG (1988). Biochemical efficacy and safety of monthly augmentation therapy for α1-antitrypsin deficiency. JAMA.

[4] Vogelmeier C, Kirlath I, Warrington S, Banik N, Ulbrich E, Du Bois RM (1997). The intrapulmonary half-life and safety of aerosolized alpha1-protease inhibitor in normal volunteers. Am J Respir Crit Care Med.

[5] McElvaney NG, Hubbard RC, Birrer P, Crystal RG, Chernick MS, Frank MM (1991). Aerosol α1 -antitrypsin treatment for cystic fibrosis. The Lancet.

[6] Hubbard RC, McElvaney NG, Sellers SE, Healy JT, Czerski DB, Crystal RG. Recombinant DNA-produced al -antitrypsin administered by aerosol augments lower respiratory tract antineutrophil elastase defenses in individuals with al-antitrypsin deficiency. The Journal of Clinical Investigation. 84:1349–54. 10.1172/JCI114305 PMC329798 2794066

[7] Hertel SP, Winter G, Friess W (2015). Protein stability in pulmonary drug delivery via nebulization. Adv Drug Deliv Rev.

[8] Flament MP, Leterme P, Burnouf T, Gayot A (1999). Influence of formulation on jet nebulisation quality of α1 protease inhibitor. Int J Pharm.

[9] Flament MP, Leterme P, Gayot A (1999). Influence of the technological parameters of ultrasonic nebulisation on the nebulisation quality of α1 protease inhibitor (α1PI). International Journal of Pharmaceutics Elsevier.

[10] Zhu W, Li L, Deng M, Wang B, Li M, Ding G (2018). Oxidation-resistant and thermostable forms of alpha-1 antitrypsin from *Escherichia coli* inclusion bodies. FEBS Open Bio.

[11] Bodier-Montagutelli E, Respaud R, Perret G, Baptista L, Duquenne P, Heuzé-Vourc’h N, et al. Protein stability during nebulization: mind the collection step! European Journal of Pharmaceutics and Biopharmaceutics. 2020;152:23–34. 10.1016/j.ejpb.2020.04.006 32289493

[12] Geller DE, Kesser KC (2010). The I-neb adaptive aerosol delivery system enhances delivery of alpha1-antitrypsin with controlled inhalation. J Aerosol Med Pulm Drug Deliv.

[13] Gaggar A, Chen J, Chmiel JF, Dorkin HL, Flume PA, Griffin R (2016). Inhaled alpha 1 -proteinase inhibitor therapy in patients with cystic fibrosis. J Cyst Fibros.

[14] Martin SL, Downey D, Bilton D, Keogan MT, Edgar J, Elborn JS (2006). Safety and efficacy of recombinant alpha1-antitrypsin therapy in cystic fibrosis. Pediatr Pulmonol.

[15] Griese M, Latzin P, Kappler M, Weckerle K, Heinzlmaier T, Bernhardt T (2006). 1-Antitrypsin inhalation reduces airway inflammation in cystic fibrosis patients. Eur Respir J.

[16] Brand P, Beckmann H, Maas Enriquez M, Meyer T, Mullinger B, Sommerer K (2003). Peripheral deposition of 1-protease inhibitor using commercial inhalation devices. Eur Respir J.

[17] Brand P, Schulte M, Wencker M, Herpich CH, Klein G, Hanna K (2009). Lung deposition of inhaled 1-proteinase inhibitor in cystic fibrosis and 1-antitrypsin deficiency. Eur Respir J.

[18] Stolk J, Tov N, Chapman KR, Fernandez P, MacNee W, Hopkinson NS (2019). Efficacy and safety of inhaled α1-antitrypsin in patients with severe α1-antitrypsin deficiency and frequent exacerbations of COPD. Eur Respir J.

[19] Franciosi AN, McCarthy C, McElvaney NG (2015). The efficacy and safety of inhaled human α-1 antitrypsin in people with α-1 antitrypsin deficiency-related emphysema. Expert Rev Respir Med.

[20] European Pharmacopoeia Online [Internet]. [cited 2023 Jan 30]. Available from: https://pheur.edqm.eu/home

[21] Bradford MM (1976). A rapid and sensitive method for the quantitation of microgram quantities of protein utilizing the principle of protein-dye binding. Anal Biochem.

[22] Schägger H, von Jagow G (1987). Tricine-sodium dodecyl sulfate-polyacrylamide gel electrophoresis for the separation of proteins in the range from 1 to 100 kDa. Anal Biochem.

[23] Bianchera A, Alomari E, Michielon A, Bazzoli G, Ronda N, Pighini G (2022). Recombinant alpha-1 antitrypsin as dry powder for pulmonary administration: a formulative proof of concept. Pharmaceutics.

[24] Miles AJ, Ramalli SG, Wallace BA (2022). DichroWeb, a website for calculating protein secondary structure from circular dichroism spectroscopic data. Protein Sci.

[25] Mazurenko S, Stourac J, Kunka A, Nedeljković S, Bednar D, Prokop Z (2018). CalFitter: a web server for analysis of protein thermal denaturation data. Nucleic Acids Res.

[26] Wewers MD, Casolaro MA, Sellers SE, Swayze SC, McPhaul KM, Wittes JT (1987). Replacement therapy for alpha 1-antitrypsin deficiency associated with emphysema. N Engl J Med.

[27] Hastedt JE, Bäckman P, Clark AR, Doub W, Hickey A, Hochhaus G (2016). Scope and relevance of a pulmonary biopharmaceutical classification system AAPS/FDA/USP Workshop March 16–17th, 2015 in Baltimore. MD AAPS Open.

[28] Kamada, Ltd. A Prospective phase III multi-center, placebo controlled, double blind study to evaluate the efficacy and safety of “Kamada-AAT for Inhalation” 80 mg per day in adult patients with congenital alpha-1 antitrypsin deficiency with moderate and severe airflow limitation (40% ≤ FEV1 ≤ 80% of predicted; FEV1/SVC ≤ 70%) [Internet]. clinicaltrials.gov; 2022 Aug. Report No.: NCT04204252. Available from: https://clinicaltrials.gov/ct2/show/NCT04204252

[29] Hubbard RC, Casolaro MA, Mitchell M, Sellers SE, Arabia F, Matthay MA, et al. Fate of aerosolized recombinant DNA-produced ai-antitrypsin: use of the epithelial surface of the lower respiratory tract to administer proteins of therapeutic importance. Medical Sciences. 1989;5. 10.1073/pnas.86.2.680 PMC286537 2783491

[30] Beasley R, Rafferty P, Holgate S (1988). Adverse reactions to the non-drug constituents of nebuliser solutions. Br J Clin Pharmacol.

[31] Snell NJC (1990). Adverse reactions to inhaled drugs. Respir Med.

[32] Zhang L, Mendoza‐Sassi RA, Wainwright C, Klassen TP. Nebulised hypertonic saline solution for acute bronchiolitis in infants. Cochrane Database Syst Rev. 2017;2017:CD006458. 10.1002/14651858.CD006458.pub4 PMC6485976 29265171

[33] Hatley RH, Byrne SM (2017). Variability in delivered dose and respirable delivered dose from nebulizers: are current regulatory testing guidelines sufficient to produce meaningful information?. Med Devices (Auckl).

[34] Ghazanfari T, Elhissi AMA, Ding Z, Taylor KMG (2007). The influence of fluid physicochemical properties on vibrating-mesh nebulization. Int J Pharm.

[35] Mc Callion ONM, Patel MJ (1996). Viscosity effects on nebulisation of aqueous solutions. Int J Pharm.

[36] Buttini F, Rossi I, Di Cuia M, Rossi A, Colombo G, Elviri L (2016). Combinations of colistin solutions and nebulisers for lung infection management in cystic fibrosis patients. Int J Pharm.

[37] Steckel H, Eskandar F (2003). Factors affecting aerosol performance during nebulization with jet and ultrasonic nebulizers. Eur J Pharm Sci.

[38] Germershaus O, Schultz I, Lühmann T, Beck-Broichsitter M, Högger P, Meinel L (2013). Insulin-like growth factor-I aerosol formulations for pulmonary delivery. Eur J Pharm Biopharm.

[39] Keller M, Tservistas M, Bitterle E, Bauer S. Aerosol characterization of alpha-1 antitrypsin after nebulization with the eFlow(R): a novel vibrating perforated membrane nebulizer. Respiratory Drug Delivery X, Boca Raton, FL, USA. 2006;733–6.

[40] Fröhlich E, Salar-Behzadi S (2021). Oral inhalation for delivery of proteins and peptides to the lungs. Eur J Pharm Biopharm.

[41] Liu X, Vanvarenberg K, Kouassi KGW, Mahri S, Vanbever R (2022). Production and characterization of mono-PEGylated alpha-1 antitrypsin for augmentation therapy. Int J Pharm.

[42] Taggart C, Cervantes-Laurean D, Kim G, McElvaney NG, Wehr N, Moss J (2000). Oxidation of either methionine 351 or methionine 358 in alpha 1-antitrypsin causes loss of anti-neutrophil elastase activity. J Biol Chem.

[43] Cipolla D, Gonda I, Shire SJ (1994). Characterization of aerosols of human recombinant deoxyribonuclease I (rhDNase) generated by jet nebulizers. Pharmaceutical Research: An Official Journal of the American Association of Pharmaceutical Scientists.

